# The potato (*Solanum tuberosum* L.) transcription factor *StPHR1* responds to low phosphate stress and regulates phosphorus accumulation

**DOI:** 10.3389/fpls.2026.1975545

**Published:** 2026-10-21

**Authors:** Feng Zhang, Huaijuan Gou, Yanlin An, Wenlun Wang, Haiyan Zhang

**Affiliations:** 1 School of Business Administration, Moutai Institute, Guizhou, China; 2 School of Food Engineering, Moutai Institute, Guizhou, China; 3 School of Resources and Environmental Engineering, Moutai Institute, Guizhou, China

**Keywords:** low-Pi stress, Pi accumulation, potato, *StPHR1*, tuber yield

## Abstract

Phosphorus is an essential macronutrient for plant growth and development. Potato is highly sensitive to inorganic phosphate (Pi) availability, yet the function and regulation of StPHR1, a putative core component of plant Pi signaling, remain poorly defined in this crop. Evolutionary analysis, subcellular localization assays, promoter activity analysis, CRISPR/Cas9-mediated gene editing, overexpression, and data-independent acquisition proteomics were used to characterize StPHR1. *StPHR1* encoded a nuclear-localized MYB-CC transcription factor, whose promoter exhibits preferential activity in root tips and vascular tissues. Loss of *StPHR1* function compromised low-Pi tolerance, reduced Pi accumulation in roots and leaves, and inhibited plant growth, whereas *StPHR1 overexpression enhanced* low-Pi tolerance and increased both tuber number and average tuber weight under conventional fertilized cultivation. Proteomic profiling showed that StPHR1-dependent Pi starvation responses involve metabolic reprogramming, membrane-lipid remodeling, redox homeostasis, and detoxification. These results identify *StPHR1* as a key regulator of Pi accumulation and allocation in potato and provide candidate targets and a molecular framework for improving low-Pi adaptation.

## 1 Introduction

Phosphorus is an essential macronutrient for plant growth and development, with central roles in nucleic acid and phospholipid biosynthesis, energy metabolism, and signal transduction (Raghothama, 1999; Zulfiqar et al., 2025). However, inorganic phosphate (Pi), the form directly available for plant uptake, is present at low concentrations in many soils. Pi readily forms insoluble complexes with soil cations such as Fe^3+^, Al^3+^, and Ca^2+^, as well as with organic matter, thereby limiting its mobility and bioavailability (Vance et al., 2003). To sustain crop yields, agricultural systems have long relied on substantial phosphate fertilizer inputs. Yet crop recovery of applied Pi within a single growing season is generally only 10%–25%. Excessive fertilizer application increases production costs and contributes to environmental degradation, particularly freshwater eutrophication (Soumya et al., 2022; Li et al., 2024). In addition, phosphate rock is a finite and non-renewable resource. Understanding how plants perceive and adapt to Pi-deficient (low Pi) conditions and developing cultivars with improved phosphorus use efficiency are therefore important priorities for sustainable agriculture.

Plants respond to low-Pi stress through coordinated morphological, physiological, and molecular adjustments collectively termed the phosphate starvation response (PSR) (Puga et al., 2024). Morphologically, Pi limitation inhibits primary root elongation while promoting lateral root and root hair proliferation, thereby increasing the absorptive surface area. Physiologically, roots exude protons and organic acids to solubilize fixed soil Pi and increase acid phosphatase and ribonuclease activities to recycle organic phosphorus. At the molecular level, high-affinity PHT1 phosphate transporters are strongly induced, reprogramming Pi uptake, translocation, and internal reallocation (Rouached et al., 2010; Poirier et al., 2022). These responses are integrated by local root-intrinsic signals and systemic signals, including phloem-mobile microRNA399 (Madison et al., 2023). Phosphorus signaling also interacts with nitrogen, sulfur, and iron signaling and with auxin- and ethylene-mediated pathways (Khan et al., 2014; Li et al., 2022). This integration enables plants to coordinate growth with multiple environmental and nutritional cues.

The PHOSPHATE STARVATION RESPONSE (PHR) family of MYB-CC transcription factors occupies a central position in plant Pi signaling (Wang et al., 2024; Puga et al., 2024). PHR1 was first characterized in *Arabidopsis*, where it binds the P1BS cis-element (GNATATNC) in target promoters through its MYB domain and activates a broad set of phosphate starvation-induced (PSI) genes (Rubio et al., 2001). *PHR1* loss-of-function mutations compromise the induction of most *PSI* genes; whereas *PHR1* overexpression causes Pi hyperaccumulation even under Pi-replete conditions (Bustos et al., 2010). The rice homolog *OsPHR2* has a comparable function; its overexpression increases shoot Pi accumulation and alters root system architecture (Zhou et al., 2008). Among OsPHR1–OsPHR4, functional redundancy is accompanied by member-specific specialization (Guo et al., 2015). PHR activity is also controlled upstream by SPX-domain proteins. Under Pi-replete conditions, SPX proteins interact with PHR in an inositol pyrophosphate (InsP)-dependent manner and repress its transcriptional activity. When Pi becomes limiting, PHR is released from this inhibitory complex and activates the PSR program (Puga et al., 2014; Wild et al., 2016). The SPX–InsP–PHR module therefore constitutes a central regulatory hub in plant Pi signaling.

Beyond their canonical roles in Pi homeostasis, PHR transcription factors regulate diverse physiological processes across plant lineages (Schmidt et al., 2025; Mangalakkadan et al., 2026). In *Arabidopsis*, PHR1 family members, including PHL1 and PHL4, act cooperatively to fine-tune the magnitude and timing of the transcriptional phosphate starvation response (Wang et al., 2023). Studies of GmPHR25 in soybean, TaPHR1 in wheat, and ZmPHR1 in maize have further linked PHR activity to Pi homeostasis and yield-related traits (Wang et al., 2013; Xue et al., 2017; Tian et al., 2024). In *Solanaceae*, the SPX–PHR module shows evidence of lineage-specific diversification. Pepper CaPHR3 enhances low-Pi tolerance by activating phosphate transporter genes (Chen et al., 2025). In tomato, SlPHL1 is induced by phosphate starvation and directly regulates anthocyanin biosynthetic genes (Wu et al., 2023), whereas silencing SlSPX1 and SlSPX2 promotes root growth and arbuscular mycorrhizal symbiosis (Singh et al., 2023). PHR1 also integrates phosphorus status with hormonal and other nutrient signals; for example, nitrogen-inducible GLK1 modulates the Pi starvation response through a PHR1-dependent mechanism (Li et al., 2022). PHR1 has additionally been implicated in iron and zinc homeostasis and in plant immune responses. Together, these findings position PHR proteins as regulatory nodes linking phosphorus nutrition to whole-plant physiological homeostasis.

Potato is the world’s fourth most important staple food crop and is highly sensitive to phosphorus availability (Nasr Esfahani et al., 2025; Hazarika et al., 2025). Its shallow root system and relatively low root hair density limit Pi acquisition, and potato often shows a strong yield response to phosphorus fertilization even in soils with high total phosphorus content (McArthur and Knowles, 1993; Iwama, 2008; Gelaye et al., 2026). In commercial production, fertilizer is frequently applied in excess to protect yield, which further reduces phosphorus use efficiency and increases environmental costs. Despite the agronomic importance of phosphorus management, the molecular function of StPHR1 in potato remains poorly characterized. To date, only one study has examined StPHR1 under combined low Pi and aluminum toxicity stress (Zhang et al., 2024). Its spatiotemporal expression, subcellular localization, downstream regulatory network, and contributions to Pi accumulation and root development remain insufficiently understood. In this study, *StPHR1* was analyzed through phylogenetic comparison, gene cloning, promoter activity analysis, and subcellular localization assays. *StPHR1* overexpression and CRISPR/Cas9-mediated knockout lines were then used to evaluate its effects on Pi accumulation, root development, and tissue-level phosphorus partitioning. Quantitative proteomics was performed to identify StPHR1-dependent low Pi-responsive proteins. These analyses establish the function of *StPHR1* in potato low-Pi adaptation and provide candidate targets for breeding phosphorus-efficient cultivars.

## 2 Materials and methods

### 2.1 Plant materials and growth conditions

Plant materials comprised potato cultivar ‘Qingshu No. 9’ (wild type, WT), two independent *StPHR1* knockout lines (*StPHR1*^ko^#27 and *StPHR1*^ko^#42), and two overexpression lines (OE-*StPHR1*#7 and OE-*StPHR1*#13). Plants were grown in a controlled chamber under a 25 °C/18 °C day/night temperature regime, a 16 h light/ 8 h dark photoperiod, 65%–70% relative humidity, and a photosynthetic photon flux density of approximately 200 μmol·m^−2^·s^−1^. Two phosphorus treatments were applied: Pi sufficiency (+Pi, 1.2 mM KH_2_PO_4_) and low-Pi stress (−Pi, 0.05 mM KH_2_PO_4_). Both media were prepared from Pi-free half-strength Murashige and Skoog (1/2 MS) medium, and the pH was adjusted to 5.8. Seedlings were grown vertically for 14 d before phenotypic and physiological measurements.

*Arabidopsis thaliana* ecotype Columbia-0 (Col-0) was used for promoter-driven GUS histochemical assays. Seeds were surface-sterilized with 75% (v/v) ethanol, sown on 1/2 MS medium, stratified at 4 °C in darkness for 2 d, and transferred to a climate chamber at 22 °C under a 16 h light/ 8 h dark photoperiod. Homozygous T_2_ transgenic seedlings were used for histochemical staining. *Nicotiana benthamiana* (*N. benthamiana*) plants were used for transient expression assays to determine the subcellular localization of StPHR1. Plants were grown at 25 °C under a 14-h light/10-h dark photoperiod. Fully expanded leaves from 4- to 6-week-old plants were infiltrated with *Agrobacterium tumefaciens* cultures, and leaf samples were collected for fluorescence observation 48–72 h after infiltration.

### 2.2 Phylogenetic tree construction

Full-length amino acid sequences of PHR homologs were retrieved from Phytozome v14, NCBI, and UniProt. The dataset comprised 37 species representing six *Solanaceae* species, 20 non-*Solanaceae* eudicots, and 11 monocots. Multiple sequence alignment was performed using ClustalW v2.1 with a gap-opening penalty of 10.0 and a gap-extension penalty of 0.1. A phylogenetic tree was constructed from the alignment using the neighbor-joining method in MEGA 10. Branch support was assessed with 1,000 bootstrap replicates, and evolutionary distances were calculated using the Poisson correction model.

### 2.3 Subcellular localization

The full-length *StPHR1* coding sequence without the stop codon was amplified by PCR and cloned into pCAMBIA1300-35S::GFP using the In-Fusion HD Cloning Kit (Takara Bio) to generate the 35S::StPHR1-GFP expression construct. The empty 35S::GFP vector served as the negative control. Recombinant constructs were introduced individually into *Agrobacterium tumefaciens* strain GV3101, and the resulting bacterial suspensions were infiltrated into the abaxial epidermis of *N. benthamiana* leaves. After 48–72 h, epidermal peels were collected from the infiltrated regions and imaged using a Leica TCS SP8 laser-scanning confocal microscope. GFP fluorescence was detected with excitation at 488 nm and emission at 507 nm. The nuclear marker 35S:NLS-mKate was excited at 587 nm, and emission was collected at 610 nm; chlorophyll autofluorescence was recorded at 680–720 nm (Dingwall et al., 1982; Tanz et al., 2013). Image acquisition and z-stack projection were performed using Leica LAS X software.

### 2.4 Cloning of the StPHR1 promoter and GUS histochemical staining

Based on the potato DM v6.1 reference annotation, an approximately 2,000-bp genomic fragment upstream of the *StPHR1* translation initiation codon (ATG) was amplified by PCR as the putative promoter region. Primers were designed to exclude annotated transposons and repetitive sequences. The amplified fragment was cloned upstream of the β-glucuronidase (GUS) reporter gene in pCAMBIA1301 to generate the Pro*StPHR1*:GUS construct. The resulting plasmid was introduced into Col-0 by *Agrobacterium tumefaciens*-mediated floral dip transformation. T_1_ seeds were screened for hygromycin resistance, and homozygous T_2_ lines were selected for histochemical assays.

Fourteen-day-old homozygous T_2_ seedlings were fixed in 90% (v/v) acetone on ice for 15 min and then incubated in GUS staining solution containing 1 mM 5-bromo-4-chloro-3-indolyl-β-D-glucuronide (X-Gluc), 50 mM sodium phosphate buffer (pH 7.0), 0.1% (v/v) Triton X-100, and 0.5 mM each of potassium ferricyanide and potassium ferrocyanide. Staining was performed at 37 °C for 12–16 h in darkness. Samples were then cleared with 70% (v/v) ethanol until the tissue background became transparent. Staining patterns were observed and photographed using a Leica M205 FA stereomicroscope; a 200-μm scale bar was applied to all micrographs.

### 2.5 Construction of CRISPR/Cas9 knockout vectors and genetic transformation

Based on the *StPHR1* genomic sequence in the potato DM v6.1 reference assembly, two single-guide RNA (sgRNA) target sites were designed in the third and fourth exons using CRISPR-P v2.0. Complementary sgRNA oligonucleotides were annealed and ligated into the pCAMBIA1300-Cas9 binary vector, which contains an OsU6 promoter for sgRNA transcription and a 35S::Cas9 expression cassette. The recombinant construct was verified by Sanger sequencing and introduced into potato cultivar ‘Qingshu No. 9’ by *Agrobacterium tumefaciens*-mediated transformation. After co-cultivation, residual Agrobacterium was eliminated with 250 mg/L cefotaxime, and transformants were selected on medium containing 50 mg/L kanamycin. Regenerated plantlets were acclimated under controlled conditions before transfer to the greenhouse.

Genomic DNA was extracted from independent regenerated lines, and fragments spanning both target sites were amplified by PCR and subjected to Sanger sequencing. Mutation genotypes and editing efficiencies were analyzed using Tracking of Indels by Decomposition (TIDE) and Inference of CRISPR Edits (ICE). Frameshift mutations were defined as insertions or deletions that disrupted the open reading frame (ORF) and introduced a premature termination codon. Putative homozygous knockout lines were identified by the absence of WT sequence peaks at both target sites.

### 2.6 Generation of overexpression lines

To generate *StPHR1* overexpressing lines, the full-length *StPHR1* coding sequence (CDS) was cloned into the pCAMBIA1300 binary vector under the control of the cauliflower mosaic virus 35S (CaMV 35S) promoter. The verified construct was introduced into *Agrobacterium tumefaciens* strain GV3101 and used to transform leaf explants of potato cultivar ‘Qingshu No. 9’. Transgenic plantlets were regenerated on kanamycin-containing medium. *StPHR1* transcript abundance in positive transformants was quantified by reverse transcription quantitative PCR (RT-qPCR). Two independent lines with stable and significantly elevated *StPHR1* expression, designated OE-*StPHR1*#7 and OE-*StPHR1*#13, were selected for subsequent experiments.

### 2.7 Analysis of growth phenotypes, Pi content, and tuber traits

After 14 d of Pi-sufficient or Pi-deficient (low Pi) treatment, whole-seedling phenotypes were documented photographically. Relative primary root length was calculated as the percentage of primary root length relative to that of the WT control. Whole-plant fresh weight was determined as the combined fresh mass of shoot and root tissues; root and shoot biomasses were not separated in this assay, precluding calculation of root-to-shoot ratios. Three biological replicates were analyzed for each treatment, with at least 15 seedlings per replicate.

Pi content was quantified using the molybdenum blue colorimetric method. Approximately 100 mg of root, stem, or leaf tissue was harvested, snap-frozen in liquid nitrogen, and ground to a fine powder. Pi was extracted with 10% (w/v) trichloroacetic acid (TCA). Following centrifugation, the supernatant was mixed with ammonium molybdate–ascorbic acid reagent for color development. Absorbance was measured at 820 nm, and Pi concentration was calculated from a KH_2_PO_4_ standard curve.

Tuber agronomic traits were measured at maturity, approximately 90 d after transplanting. The tuber production trial was conducted in pots under conventional fertilized cultivation (Pi-sufficient conditions), and no low-Pi treatment was applied to tuber-bearing plants. Ten plants were randomly selected from each line to determine tuber number per plant and average fresh weight per tuber; only tubers with a diameter of at least 1 cm were counted. Fresh weight was measured using an electronic balance with a precision of 0.01 g. Data are presented as mean ± standard deviation (SD).

### 2.8 Proteomics sample preparation and DIA quantification

The quantitative proteomic experiment included six sample groups derived from three genotypes (WT, OE-*StPHR1*#7, and *StPHR1*^ko^#27) under two phosphorus regimes (+Pi and −Pi), with three biological replicates per group (18 samples in total). Seven pairwise comparisons were used for differential expression analysis: OE-*StPHR1*#7 −Pi versus +Pi, OE-*StPHR1*#7 versus WT under −Pi, *StPHR1*^ko^#27 versus WT under −Pi, OE-*StPHR1*#7 versus WT under +Pi, WT −Pi versus +Pi, *StPHR1*^ko^#27 versus WT under +Pi, and *StPHR1*^ko^#27 −Pi versus +Pi. The first six comparisons are presented in the main figures (**Figures 1**, **2**), and the seventh is shown in **Supplementary Figure S2**. One representative line per genotype was selected for proteomic profiling on the basis of the molecular and phenotypic characterization described in Sections 3.3 and 3.4; both independent knockout lines carried frameshift alleles and showed concordant low-Pi-hypersensitive phenotypes (**Figure 3**), and both overexpression lines showed comparable *StPHR1* transcript accumulation and enhanced low-Pi tolerance (**Figure 4**). Root tissues (~200 mg per sample) were harvested from 14-day-old seedlings at the end of the 14-d +Pi or −Pi treatment described in Section 2.7, snap-frozen in liquid nitrogen, and stored at −80 °C until processing. Protein extraction and tryptic digestion followed a standard data-independent acquisition (DIA) proteomics workflow. Briefly, root tissues were lysed in buffer containing 8 M urea, 100 mM NH_4_HCO_3_, and a protease inhibitor cocktail. After centrifugation at 12,000 × g for 5 min at 4 °C, the supernatant was collected, and proteins were precipitated with acetone. Protein concentration was determined using the Pierce BCA Protein Assay Kit. For each sample, 50 μg of total protein was reduced with 56 mM dithiothreitol (DTT) at 56 °C for 1 h and alkylated with 110 mM iodoacetamide for 30 min at room temperature in darkness. The urea concentration was diluted below 2 M with 100 mM NH_4_HCO_3_. Trypsin (Trypsin Gold, Promega) was added at an enzyme-to-protein mass ratio of 1:50, and digestion was performed overnight at 37 °C with shaking. Digestion was terminated by adding trifluoroacetic acid (TFA) to a final concentration of 0.5% (v/v). Peptides were desalted using ZipTip C18 columns (Merck Millipore), vacuum-dried, and stored at −20 °C until mass spectrometric analysis.

**Figure 1:**
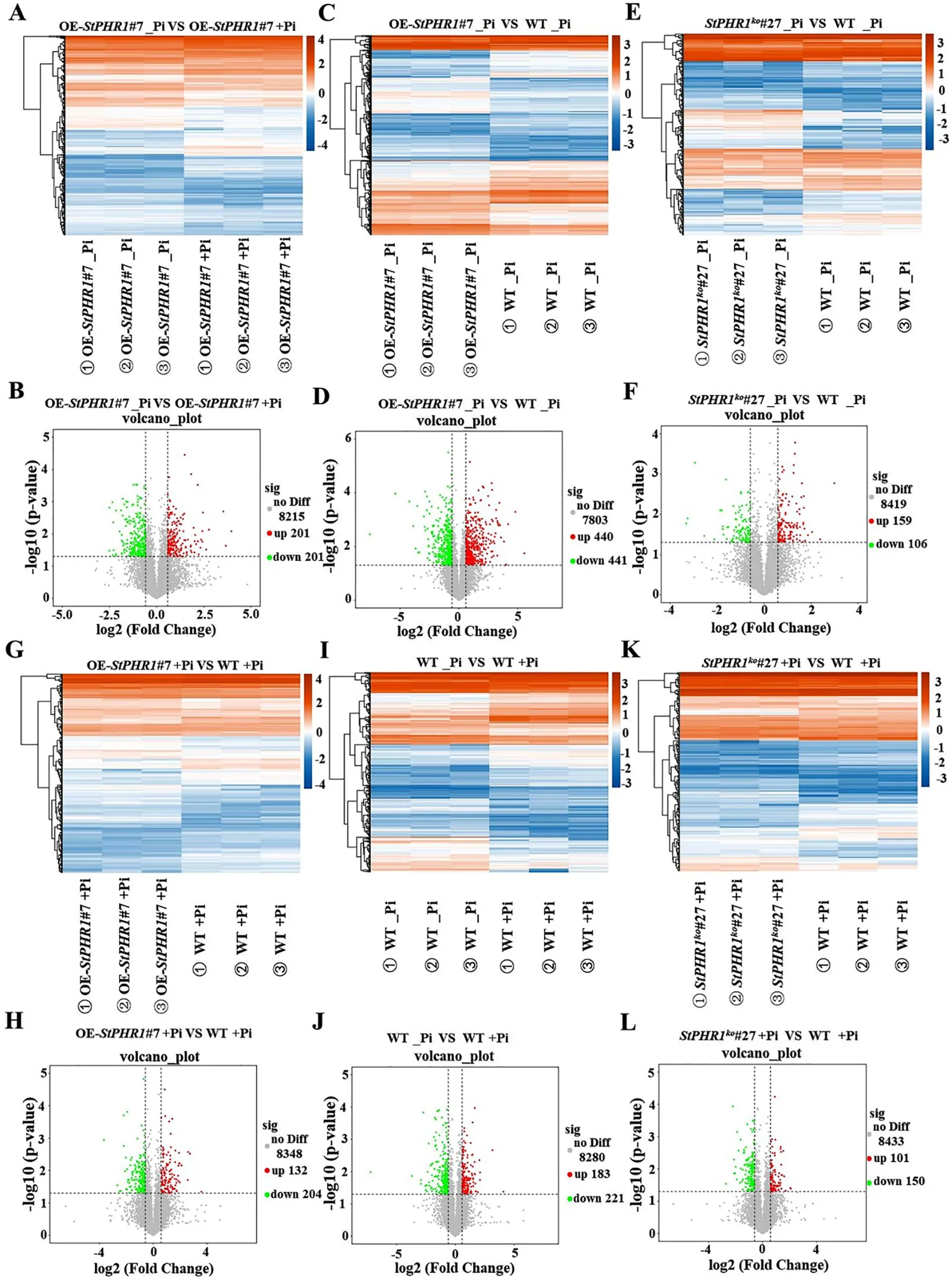
Hierarchical clustering and volcano plot analyses of differentially expressed proteins. **(A, C, E, G, I, K)** Hierarchical clustering heatmaps of differentially expressed proteins. Orange and blue represent increased and decreased protein abundance, respectively, with color intensity scaled to relative abundance. Biological replicates from each group cluster tightly together, indicating strong within-group reproducibility. **(B, D, F, H, J, L)** Volcano plots of differentially expressed proteins for each pairwise comparison. The x-axis shows log_2_-transformed fold change, and the y-axis shows −log_10_-transformed P values. Red and green dots denote significantly upregulated and downregulated proteins, respectively; gray dots indicate proteins without significant abundance changes. The numbers of upregulated, downregulated, and non-significant proteins are annotated in each plot. Six of the seven pairwise comparisons (see **Figure 8B**) are shown here (the seventh, *StPHR1*ko#27 −Pi versus +Pi, is presented in **Supplementary Figure S2**): **(A, B)** OE-*StPHR1*#7 under low-Pi versus Pi-sufficient conditions; **(C, D)** OE-*StPHR1*#7 versus WT under low-Pi conditions; **(E, F)** *StPHR1*^ko^#27 versus WT under low-Pi conditions; **(G, H)** OE-*StPHR1*#7 versus WT under Pi-sufficient conditions; **(I, J)** WT under low-Pi versus Pi-sufficient conditions; and **(K, L)** *StPHR1*^ko^#27 versus WT under Pi-sufficient conditions.

**Figure 2:**
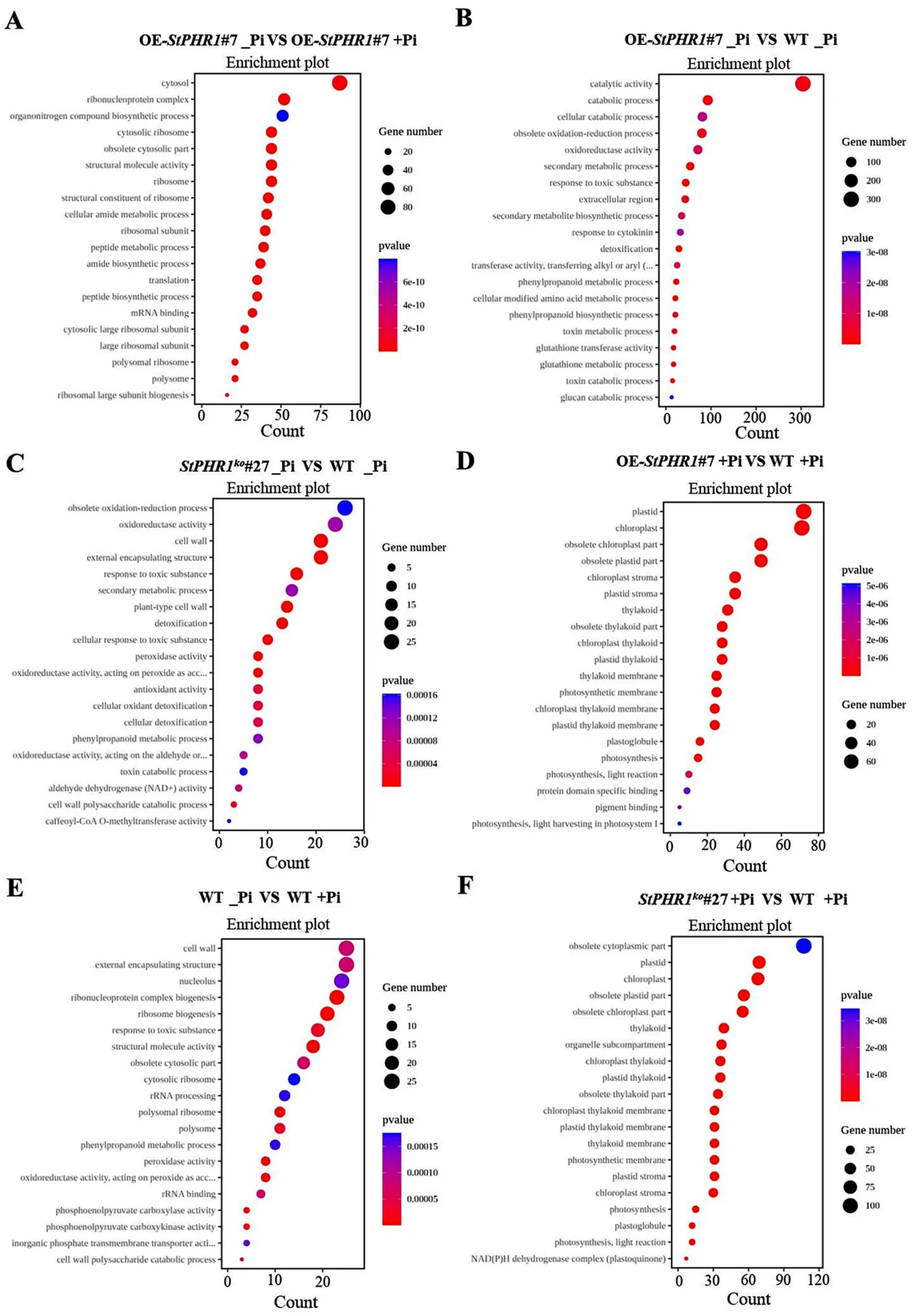
GO functional enrichment analysis of differentially expressed proteins across six pairwise comparisons (the seventh comparison, *StPHR1*ko#27 −Pi versus +Pi, is shown in **Supplementary Figure S3**). **(A)** Low Pi (−Pi) versus Pi-sufficient (+Pi) comparison in OE-*StPHR1*#7. **(B)** OE-*StPHR1*#7 versus WT under low-Pi conditions. **(C)** *StPHR1*^ko^#27 versus WT under low-Pi conditions. **(D)** OE-*StPHR1*#7 versus WT under Pi-sufficient conditions. **(E)** Low Pi (−Pi) versus Pi-sufficient (+Pi) comparison in WT. **(F)** *StPHR1*^ko^#27 versus WT under Pi-sufficient conditions. Each dot represents one enriched GO term. Dot diameter is positively correlated with the number of differentially expressed proteins assigned to the term. The color gradient from blue to red represents increasing enrichment significance (decreasing P values), with red indicating the highest significance. The x-axis shows the number of enriched proteins assigned to each GO term.

**Figure 3:**
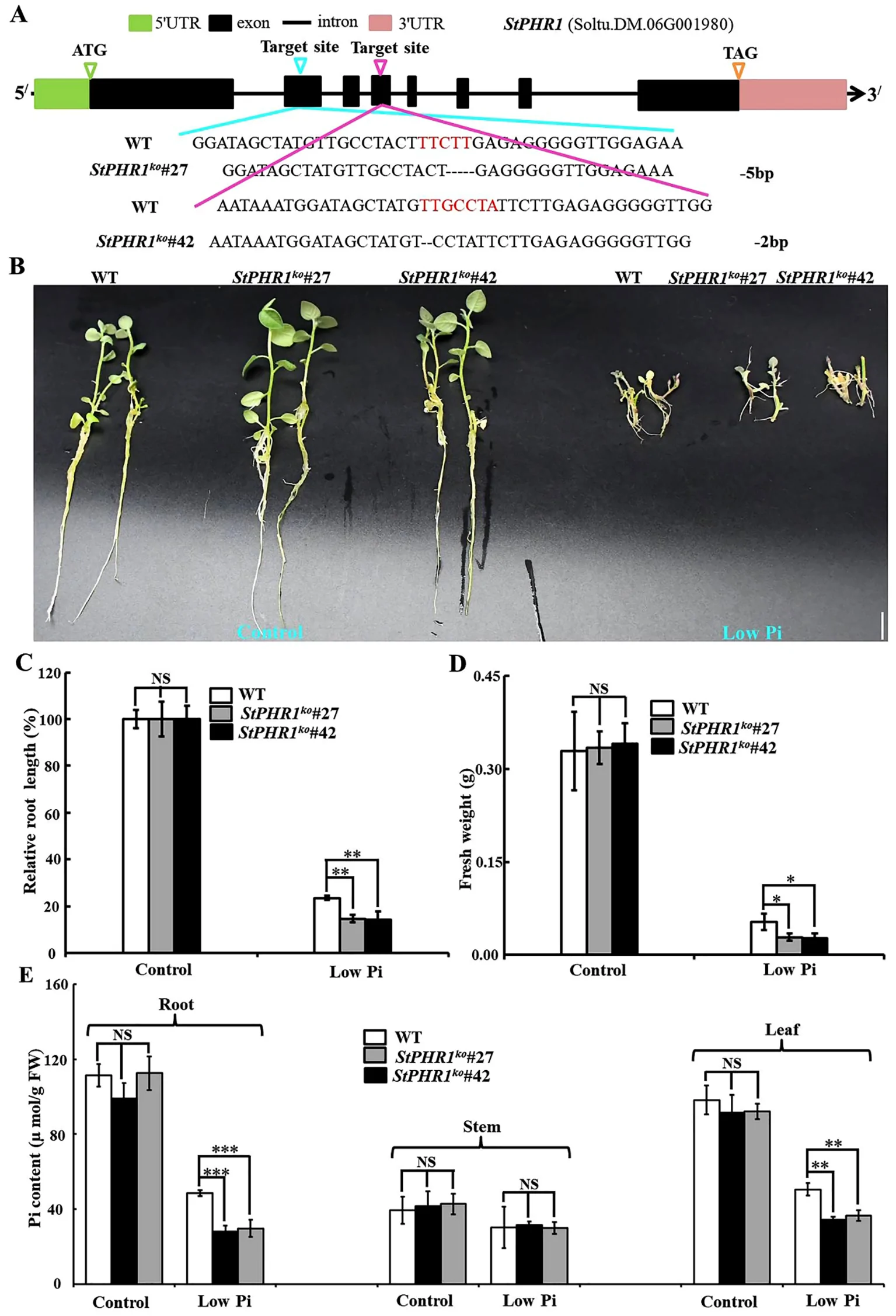
Generation of *StPHR1* knockout mutants and their growth phenotypes and phosphorus accumulation under low-phosphate stress. **(A)** Schematic representation of the potato *StPHR1* (Soltu.DM.06G001980) gene structure and CRISPR/Cas9 target sites. Green rectangles, black rectangles, black lines, and pink rectangles denote the 5′ UTR, exons, introns, and 3′ UTR, respectively; blue arrows mark the two editing target sites. Sequence alignments of the target regions from WT and the homozygous knockout lines *StPHR1*^ko^#27 and *StPHR1*^ko^#42 are shown below. The two lines carry 5-bp and 2-bp deletions, respectively, both of which introduce frameshift mutations in the open reading frame. **(B)** Growth phenotypes of WT and knockout seedlings under Pi-sufficient (Control) and Pi-deficient (Low Pi) conditions. Scale bar = 1 cm. **(C)** Relative primary root length of each line. **(D)** Whole-plant fresh weight of each line. **(E)** Inorganic Pi contents in root, stem, and leaf tissues. NS, not significant; *P < 0.05; **P < 0.01; ***P < 0.001.

**Figure 4:**
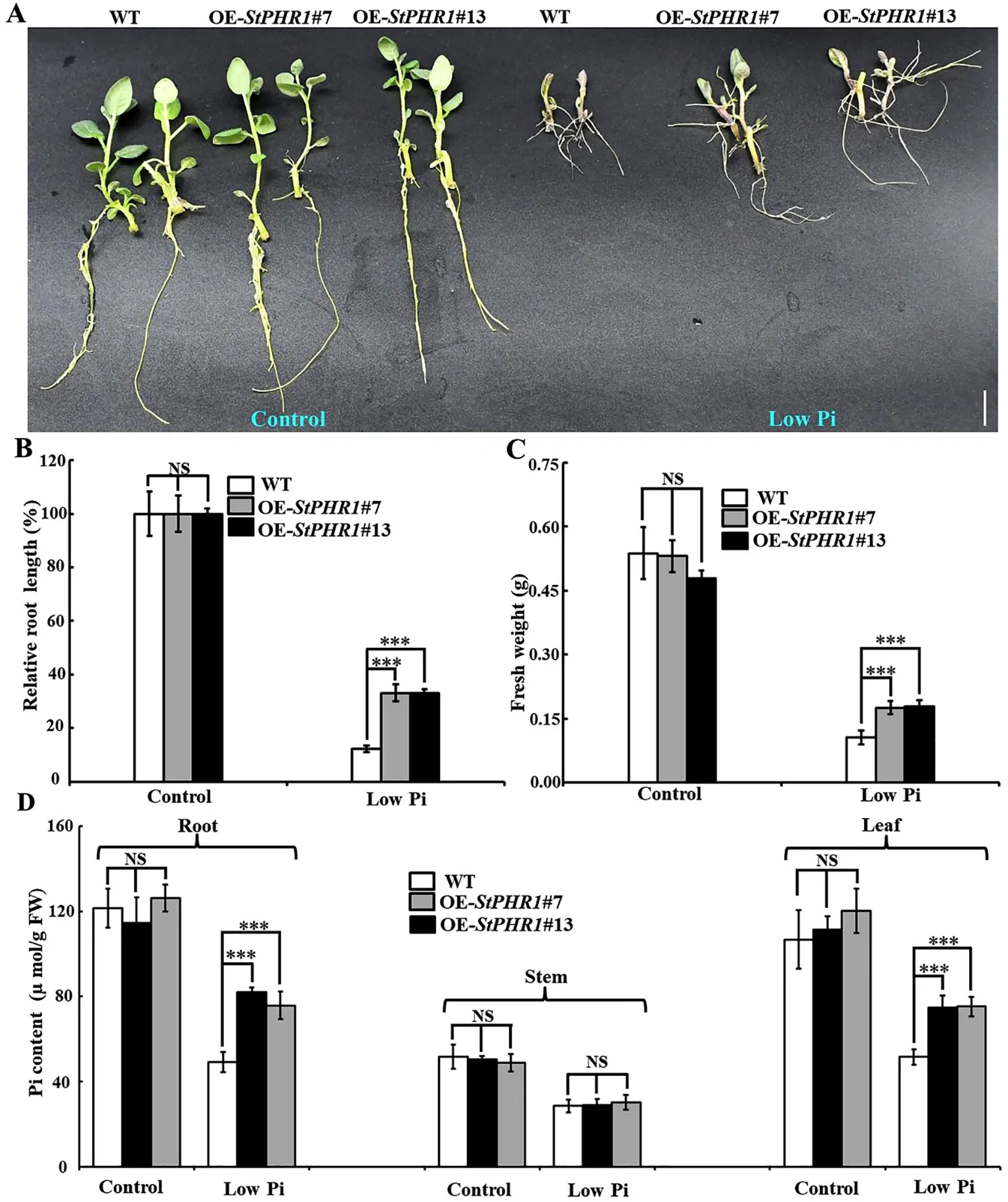
Overexpression of *StPHR1* enhances low-phosphate tolerance in potato seedlings. **(A)** Growth phenotypes of WT and two independent *StPHR1* overexpression lines (OE-*StPHR1*#7 and OE-*StPHR1*#13) under control and low-Pi conditions. Scale bar = 1 cm. **(B)** Relative primary root length across all lines. **(C)** Whole-plant fresh weight across all lines. **(D)** Pi contents in root, stem, and leaf tissues. NS, not significant; *P < 0.05; **P < 0.01; ***P < 0.001.

Chromatographic separation was performed on a Vanquish Neo ultra-high-performance liquid chromatography system (Thermo Fisher Scientific). Peptides were loaded onto a trap column (300 μm × 5 mm, 5-μm particle size, 100-Å pore size) and separated on an analytical column (150 μm × 150 mm, 2-μm particle size, 100-Å pore size). Mobile phase A was 0.1% (v/v) formic acid in water, and mobile phase B was 0.1% (v/v) formic acid in 80% acetonitrile. The flow rate was 1.8 μL/min, and the total gradient elution time was 60 min. Mass spectrometric analysis was conducted on a Thermo Scientific Orbitrap Astral high-resolution mass spectrometer operated in DIA mode. MS1 full scans were acquired at a resolution of 240,000 with automatic gain control (AGC) set to Custom mode. DIA scans covered the full mass-to-charge range using 2-Da isolation windows, and the normalized HCD collision energy was set to 25%.

Raw mass spectrometry data were processed for protein identification and quantification using DIA-NN v1.8.1. An in silico spectral library was generated with the built-in deep-learning algorithm of DIA-NN using the potato reference proteome from UniProt (44,851 sequences). The match-between-runs (MBR) function was enabled to improve peptide identification coverage. A false discovery rate (FDR) threshold of 1% was applied at both the precursor and protein levels. In total, 72,898 peptides and 8,684 proteins were identified, of which 7,927 proteins were quantified. Differentially expressed proteins (DEPs) were defined by Student’s t-test (P < 0.05) and fold change (FC > 1.5 or FC < 0.667) for comparisons with biological replicates; FC alone was used for comparisons without replicates. No multiple-testing correction was applied at the DEP-calling step; this nominal P-value-plus-fold-change strategy is commonly used in DIA proteomics with limited replication to preserve sensitivity. To control false positives at the level of functional interpretation, all downstream GO and KEGG enrichment analyses were required to pass Benjamini–Hochberg correction (adjusted P < 0.05), and proteomic conclusions are drawn at the pathway level rather than for individual proteins.

### 2.9 Bioinformatics analysis

Following log_2_ transformation of the global protein abundance matrix, sample reproducibility was evaluated by calculating Pearson correlation coefficients among biological replicates. Hierarchical clustering heatmaps and volcano plots were generated using the R packages pheatmap (v1.0.12) and ggplot2 (v3.4.0), respectively. K-means clustering of the full protein expression dataset was performed using the kmeans function in the R stats package. Proteins were partitioned into nine clusters (k = 9) based on the elbow point of within-cluster variance and biological interpretability, and expression trends for each cluster were visualized with ggplot2.

Gene Ontology (GO) annotation and enrichment analysis of differentially expressed proteins (DEPs) were performed using the R package clusterProfiler (v4.2.2), with the UniProt-GOA potato annotation set as the reference. Enrichment significance was determined using a hypergeometric test, with Benjamini–Hochberg correction and an adjusted P < 0.05 threshold. WEGO-style GO classification bar plots were generated using custom R scripts, and terms were grouped into biological process (BP), cellular component (CC), and molecular function (MF). Kyoto Encyclopedia of Genes and Genomes (KEGG) pathway enrichment analysis was performed in parallel using the KEGG Orthology (KO) database, and the 20 most significantly enriched pathways were retained for visualization.

Weighted gene co-expression network analysis (WGCNA) was performed using the R package WGCNA (v1.72.5). The input matrix comprised log_2_-normalized abundance values for all quantified proteins. The soft-thresholding power was selected to satisfy the scale-free topology criterion, with a goodness-of-fit R^2^ threshold of 0.85. The topological overlap matrix (TOM) was converted to a dissimilarity matrix (1 − TOM) for hierarchical clustering. Modules were detected with a minimum module size of 30, and highly similar modules were merged using a mergeCutHeight threshold of 0.25. Pearson correlation coefficients between module eigengenes and sample traits (genotype and phosphorus treatment), together with the corresponding P values, were visualized as a heatmap.

### 2.10 Statistical analysis

All quantitative data are presented as mean ± standard deviation (SD). Pairwise comparisons between two groups were performed using Student’s t-test. For comparisons among multiple groups, one-way analysis of variance (ANOVA) was followed by Tukey’s honestly significant difference (HSD) *post hoc* test. For tuber trait analysis, Duncan’s multiple range tests were used. Statistical significance was defined as *P < 0.05, **P < 0.01, and ***P < 0.001; NS denotes no significant difference. Statistical analyses and figure and table preparation were performed using Microsoft Excel 2019 and R v4.3.0.

## 3 Results

### 3.1 *StPHR1* is evolutionarily conserved in Solanaceae and localizes to the nucleus

To determine the evolutionary position and sequence conservation of potato StPHR1, full-length amino acid sequences of PHR homologs from 37 plant species were analyzed using the neighbor-joining method. The dataset comprised six *Solanaceae* species, 20 non-*Solanaceae* eudicots, and 11 monocots (**Figure 5A**). Four major clades were resolved. All *Solanaceae* PHR1 homologs formed a well-supported monophyletic clade (Clade I), within which StPHR1 was most closely related to SlPHR1 from tomato and CaPHR1 from pepper. The remaining dicot homologs were assigned to Clades II and III, whereas all monocot proteins clustered in Clade IV. This separation reflects evolutionary divergence between monocot and dicot PHR lineages.

**Figure 5:**
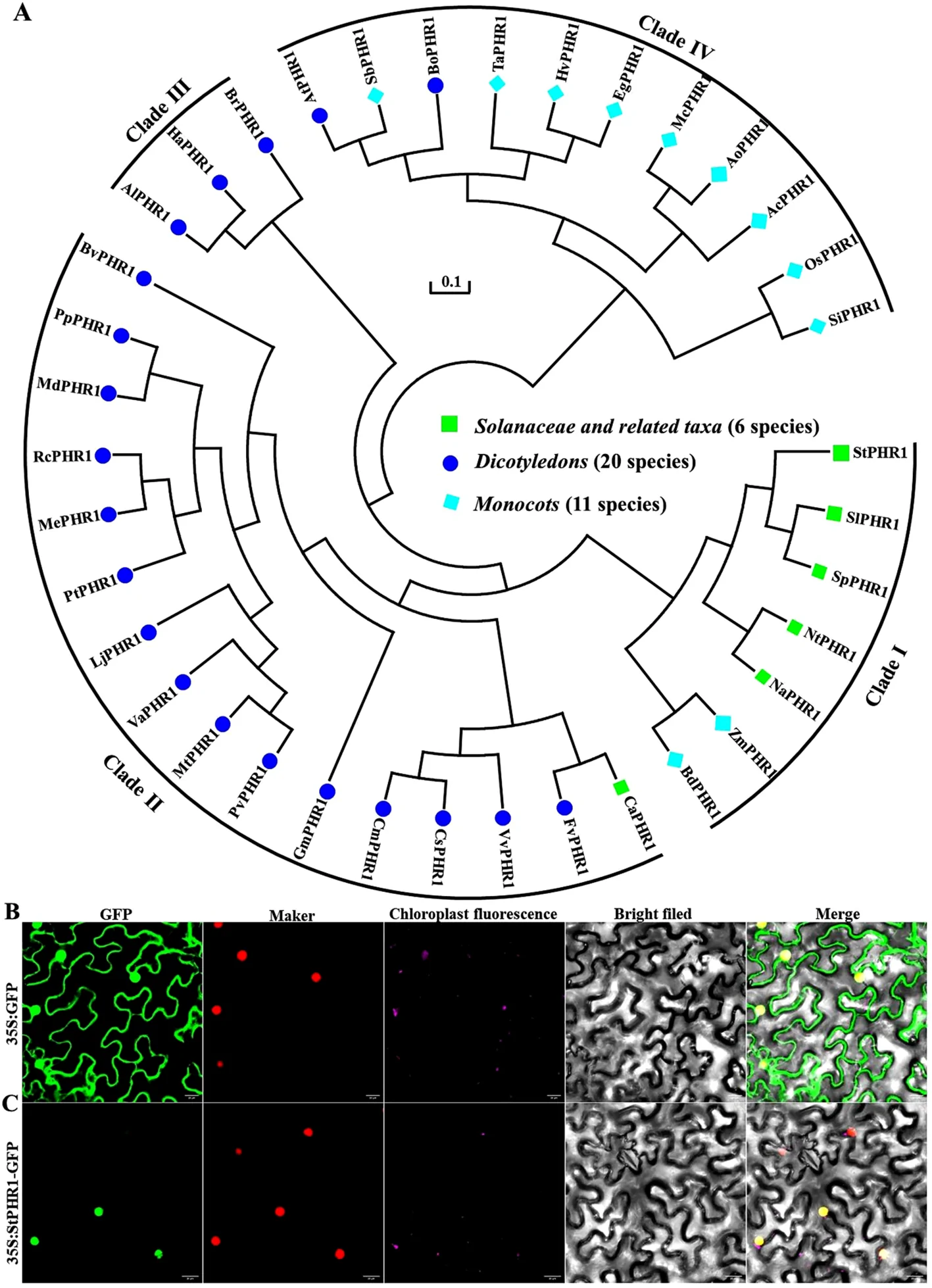
Phylogenetic analysis and subcellular localization of StPHR1. **(A)** Phylogenetic tree of PHR homologs from 37 plant species, including Solanaceae taxa, other eudicots, and monocots. The tree was constructed using the neighbor-joining method based on full-length amino acid sequences. All homologs were resolved into four clades. *Solanaceae*, other eudicot, and monocot sequences are indicated by green squares, dark blue circles, and light blue diamonds, respectively. The scale bar represents amino acid substitutions per site. **(B)** Subcellular localization analysis by transient expression in *N. benthamiana* leaf epidermal cells. The 35S::GFP empty-vector control showed diffuse fluorescence throughout the cell, without specific subcellular enrichment. **(C)** The 35S:StPHR1-GFP fusion protein localized exclusively to the nucleus, and its green fluorescence fully colocalized with the nuclear marker. No overlap with chloroplast autofluorescence was detected, and no cytosolic free GFP fluorescence was observed for the fusion protein. For **(B, C)**, images from left to right show GFP fluorescence, nuclear marker fluorescence, chlorophyll autofluorescence, bright-field images, and merged images. Scale bar = 20 μm.

The subcellular localization of StPHR1 was examined by transient expression in *N. benthamiana* leaf epidermal cells. In cells expressing the empty-vector control (35S::GFP), green fluorescence was distributed throughout the cytoplasm, plasma membrane, and nucleus (**Figure 5B**). By contrast, 35S::StPHR1-GFP fluorescence was restricted to the nucleus and fully colocalized with the nuclear marker NLS-mKate; no overlap with chloroplast autofluorescence was detected (**Figure 5C**). This nuclear localization pattern is consistent with that of characterized PHR homologs in *Arabidopsis* and rice (Zhou et al., 2008; Puga et al., 2014; Meng et al., 2025).

### 3.2 The *StPHR1* promoter is preferentially active in root tissues

To characterize the spatial activity of the *StPHR1* promoter, a Pro*StPHR1*:GUS reporter construct was introduced into *Arabidopsis* by *Agrobacterium tumefaciens*-mediated transformation, and homozygous T_2_ seedlings were examined by histochemical staining. GUS activity driven by the *StPHR1* promoter showed clear tissue specificity (**Figure 6**). In cotyledons, faint staining was restricted to leaf veins and adjacent mesophyll cells, indicating low promoter activity in shoot tissues (**Figures 6A, B**). In roots, strong GUS activity extended along the primary root from the root–hypocotyl transition zone toward the tip. Signal intensity was highest in the root apical meristem and elongation zone, whereas staining in the mature root zone formed a continuous band restricted to vascular tissues (**Figures 6C–E**). The root-preferential activity of the *StPHR1* promoter indicates that *StPHR1* is predominantly transcribed in tissues involved in Pi acquisition.

**Figure 6:**
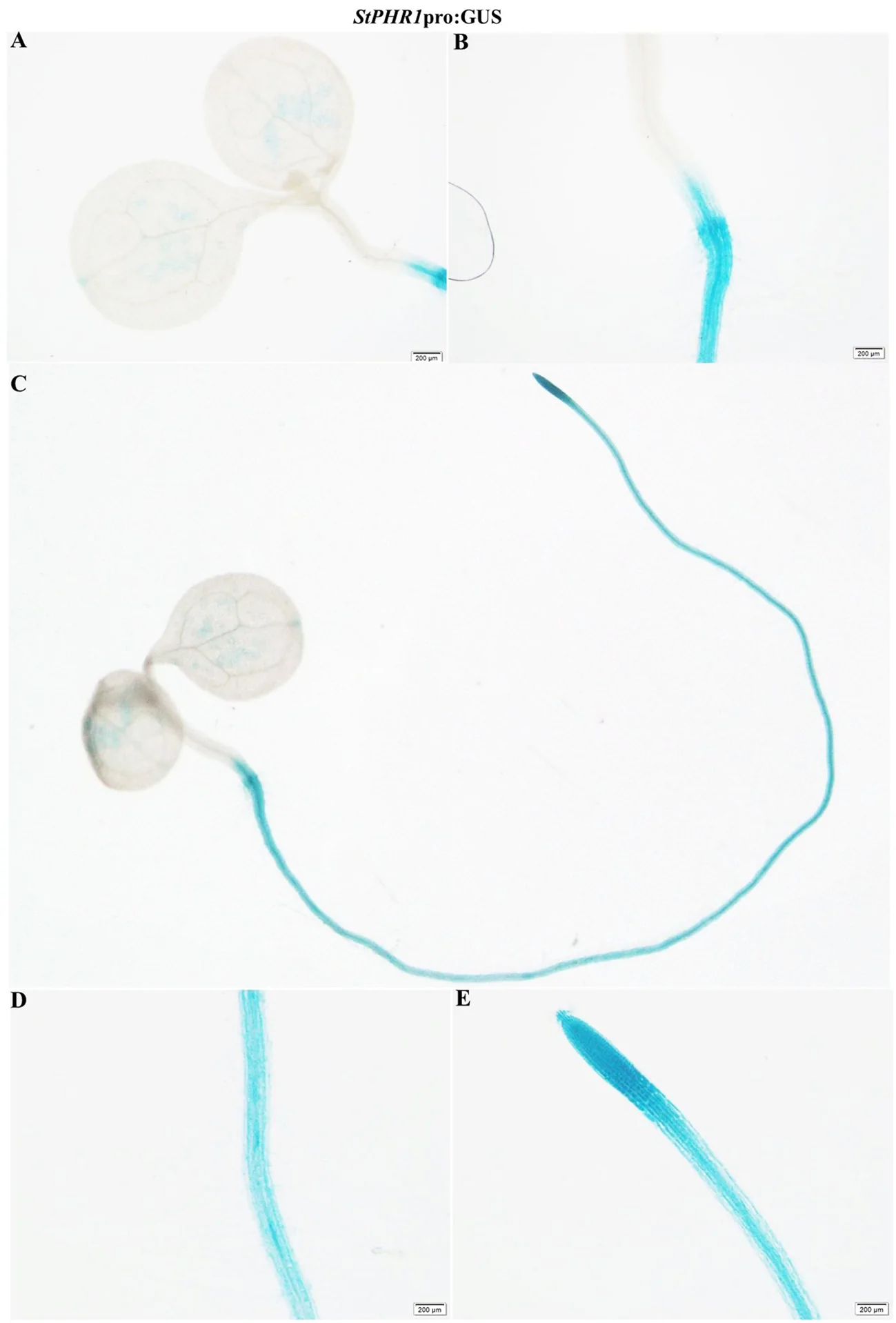
Activity pattern of the *StPHR1* promoter in transgenic *Arabidopsis*. **(A)** Histochemical GUS staining of whole transgenic seedlings. Faint blue staining was visible in cotyledon veins, with a stronger signal at the root–hypocotyl junction. **(B)** Close-up view of the root–hypocotyl transition zone. **(C)** Whole-seedling view showing GUS activity along the primary root. **(D)** Magnified view of the mature primary root zone. GUS staining showed a banded distribution restricted to vascular tissues. **(E)** Close-up view of the root tip. The strongest GUS signal was detected in the root apical meristem and elongation zone. Scale bars = 200 μm.

### 3.3 *StPHR1* knockout lines are hypersensitive to low-Pi stress

*A StPHR1-specific CRISPR/Cas9 construct was generated with two sgRNA target sites in exons 3 and 4. Following Agrobacterium tumefaciens*-mediated transformation and antibiotic selection, regenerated plants were genotyped by PCR amplification and Sanger sequencing of the target regions. Two independent homozygous knockout lines, designated *StPHR1*^ko^#27 and *StPHR1*^ko^#42, were selected for further analysis. Sequence verification identified 5-bp and 2-bp deletions at the target sites in the two lines, respectively. Both deletions caused frameshifts that introduced premature termination codons and were predicted to produce truncated, non-functional proteins (**Figure 3A**). Under Pi-sufficient conditions, plant morphology and root architecture did not differ visibly between the knockout lines and WT. Relative primary root length, whole-plant fresh weight, and Pi contents in root, stem, and leaf tissues were also comparable across genotypes (**Figures 3B–E**). Thus, loss of *StPHR1* did not disrupt seedling growth or basal Pi homeostasis when phosphorus was abundant. Under low-Pi stress, all lines showed growth inhibition, but the phenotype was markedly more severe in the knockout lines. Leaves became chlorotic and crinkled, and root elongation was strongly impaired. Relative root length was reduced by more than 20% compared with WT, and whole-plant fresh weight was approximately 60% of the WT value; both differences were highly significant (P < 0.001) (**Figures 3B–E**). These reverse-genetic data indicate that StPHR1 is required for normal low-Pi tolerance in potato seedlings. All knockout seedlings remained alive throughout the 14-d low-Pi treatment, although their growth was strongly inhibited (**Figure 3B**).

Under low-Pi stress, root and leaf Pi contents declined sharply in all lines relative to Pi-sufficient controls, and the reduction was significantly greater in the *StPHR1* knockout lines than in WT. Root Pi content was 30% lower and leaf Pi content was reduced by more than 25% compared with WT under low-Pi conditions, whereas stem Pi content did not differ significantly among genotypes (**Figure 3E**). This tissue-specific pattern indicates that loss of StPHR1 primarily affected Pi accumulation in roots and leaves, whereas stem Pi content was less affected (**Figure 3E**).

### 3.4 *StPHR1* overexpression enhances low-Pi tolerance and promotes tuber production

Under Pi-sufficient conditions, plant morphology, relative primary root length, and whole-plant fresh weight did not differ significantly between the two independent *StPHR1* overexpression lines (OE-*StPHR1*#7 and OE-*StPHR1*#13) and WT. Pi contents in root, stem, and leaf tissues were also comparable across genotypes (**Figure 4**), indicating that *StPHR1* overexpression did not disrupt basal growth or Pi homeostasis under phosphorus-replete conditions. Low-Pi stress inhibited root elongation and reduced shoot biomass in all lines, but the inhibition was significantly attenuated in the overexpression lines. Compared with WT, these lines showed a >30% increase in relative root length and an approximately 40% increase in whole-plant fresh weight (P < 0.001). Root and leaf Pi contents were approximately 35% and 28% higher than in WT, respectively, whereas stem Pi content did not differ significantly among genotypes (**Figure 4**). Together with the knockout phenotypes described above, these data define a consistent phenotypic gradient—knockout < WT < overexpression—for relative root length, whole-plant fresh weight, and tissue Pi content under low Pi (**Figures 3**, **4**).

At maturity, both tuber numbers per plant and average tuber fresh weight were significantly higher in the *StPHR1* overexpression lines than in WT under standard cultivation conditions. Tuber number per plant more than doubled, and average tuber fresh weight increased by approximately 40%, indicating that the higher tuber yield resulted from both increased tuber set and larger individual tubers (**Figure 7**). Because the tuber trial was conducted under conventional fertilized (Pi-sufficient) cultivation, these phenotypes reflect the effect of *StPHR1* overexpression under standard nutrient supply rather than a specific adaptation to low Pi.

**Figure 7:**
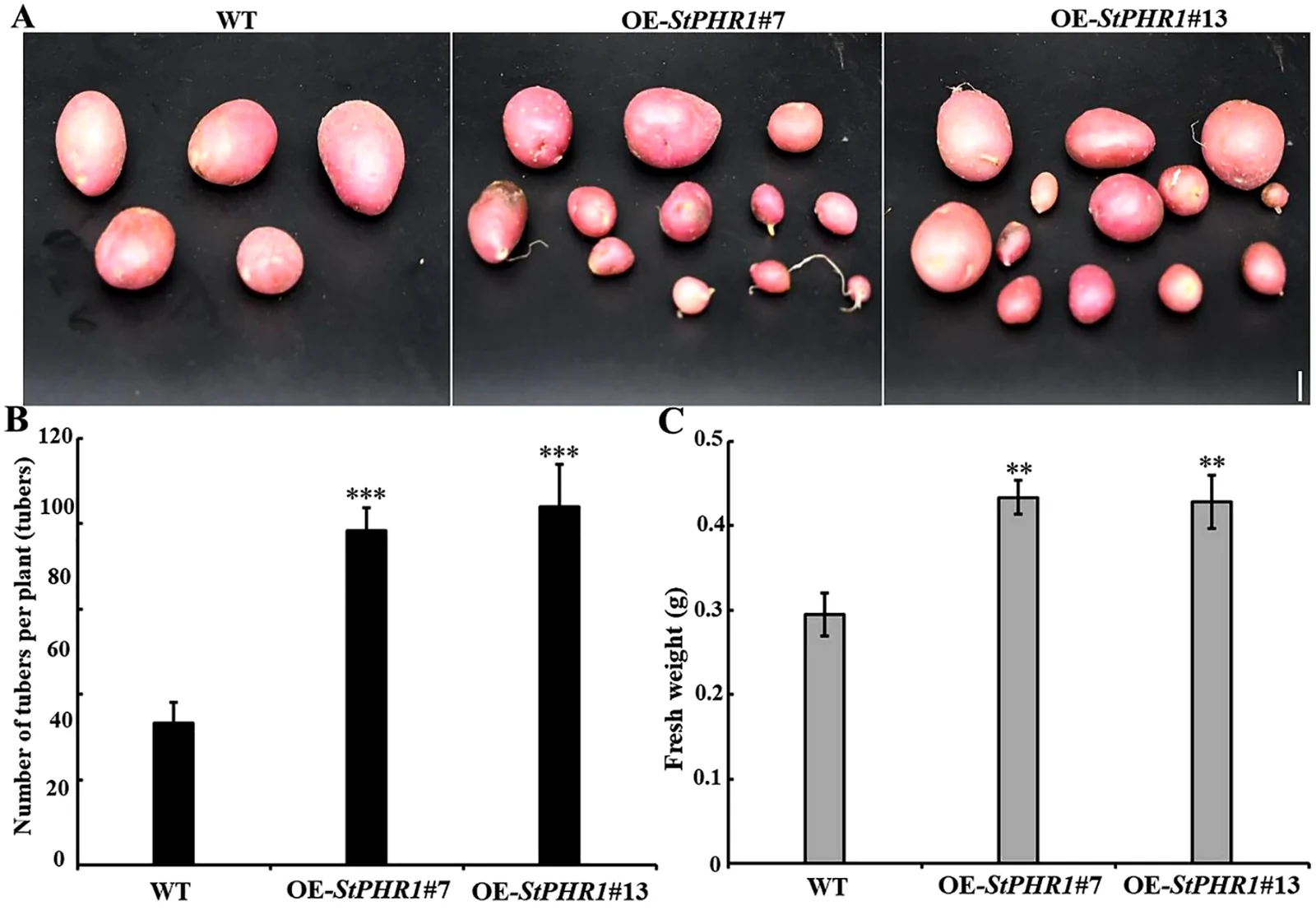
Overexpression of *StPHR1* increases tuber number and mean tuber fresh weight in potato. **(A)** Tuber phenotypes of WT and two independent *StPHR1* overexpression lines (OE-*StPHR1*#7 and OE-*StPHR1*#13) at maturity under conventional cultivation conditions. Scale bar = 1 cm. **(B)** Tuber number per plant. **(C)** Mean fresh weight per tuber. Data were analyzed using Duncan’s multiple range test. *P < 0.05; **P < 0.01; ***P < 0.001.

### 3.5 Quantitative proteomic quality assessment and identification of differentially expressed proteins

Following log_2_ transformation of global protein abundance values, abundance distributions were visualized using violin plots. Profiles were highly consistent across sample groups, with comparable medians and interquartile ranges and no evidence of a global distribution shift (**Figure 8A**). These results indicate that protein quantification was stable and comparable across genotypes, phosphorus treatments, and biological replicates. Sample-to-sample Pearson correlation analysis further supported the high data quality (**Supplementary Figure S1**). Within each treatment group, pairwise correlation coefficients among the three biological replicates exceeded 0.90, and several replicate pairs exceeded 0.95, demonstrating strong within-group reproducibility. Correlation coefficients varied among genotype–treatment combinations; lower values were observed between low Pi and Pi-sufficient treatments and between overexpression and knockout lines, consistent with treatment- and genotype-dependent proteomic effects.

**Figure 8:**
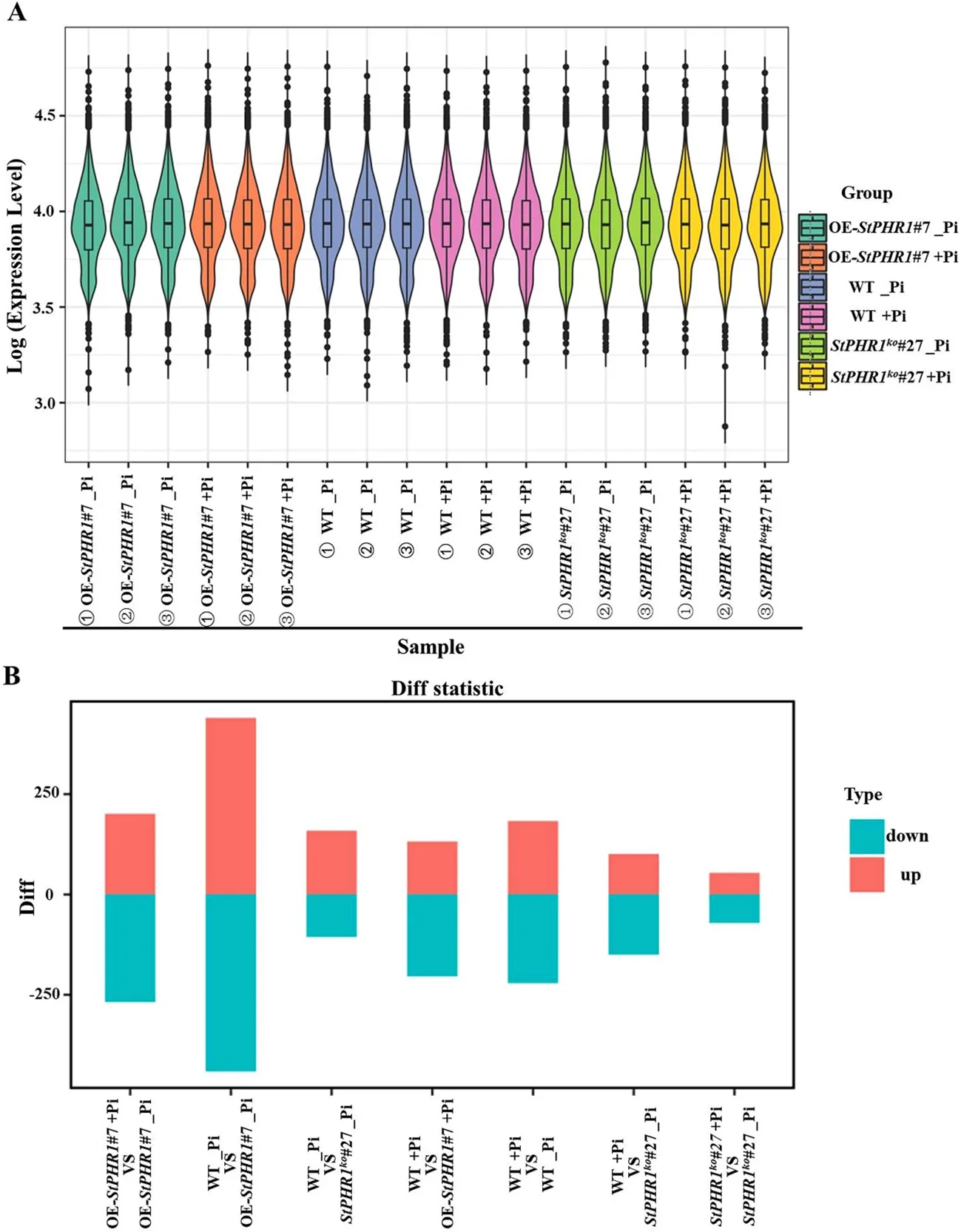
Label-free quantitative proteomic analyses of potato seedlings from different genotypes under Pi-sufficient (+Pi) and low Pi (−Pi) conditions. **(A)** Violin plots showing the global distribution of protein abundance in each sample. The y-axis represents log_2_-transformed protein abundance. The plots illustrate overall abundance patterns across samples and within-group reproducibility among biological replicates. Each group contains three biological replicates, and distinct colors denote different genotype–treatment combinations. **(B)** Stacked bar chart of differentially expressed proteins (DEPs) across seven pairwise comparisons, six of which are presented in **Figures 1** and **2**, with the seventh (*StPHR1*^ko^#27 −Pi versus +Pi) shown in **Supplementary Figure S2**. Blue and red indicate significantly downregulated and upregulated proteins, respectively, showing the total number of DEPs and the direction of regulation in each comparison.

Differentially expressed proteins (DEPs) were identified across seven pairwise comparisons—six presented in the main figures and the seventh (*StPHR1*^ko^#27 −Pi versus +Pi) shown in **Supplementary Figure S2**—and the numbers of upregulated and downregulated proteins in each comparison were summarized in a stacked bar chart (**Figure 8B**). In low-Pi versus Pi-sufficient comparisons within each genotype, the *StPHR1* overexpressing line showed the largest total number of DEPs, with a higher proportion of upregulated than downregulated proteins. WT showed an intermediate number of DEPs, whereas the *StPHR1* knockout line exhibited the fewest DEPs in response to Pi deficiency. This trend indicates that loss of *StPHR1* substantially dampens the global proteomic response to altered Pi availability. In cross-genotype comparisons under the same Pi regime, more DEPs were detected between the overexpression line and WT under low Pi than under Pi-sufficient conditions, suggesting that the regulatory effect of StPHR1 is stronger during phosphorus limitation.

### 3.6 Clustering and volcano plot analyses reveal *StPHR1* dependent low-Pi responses

Hierarchical clustering and volcano plot analyses were performed for DEPs across the pairwise comparisons. In the hierarchical clustering heatmaps, biological replicates from each treatment group formed independent and highly concordant clusters, further confirming the reproducibility of the quantitative proteomic data (**Figures 1A, C, E, G, I, K**; **Supplementary Figure S2A**). Volcano plot analysis revealed substantial variation in DEP numbers among comparisons. The comparison between the *StPHR1* overexpressing line and WT under low-Pi stress yielded the largest DEP set, with 440 upregulated and 441 downregulated proteins. By contrast, only 54 upregulated and 71 downregulated proteins were detected in the low-Pi versus Pi-sufficient comparison of the *StPHR1* knockout line, far fewer than in the corresponding WT and overexpression comparisons (**Figures 1B, D, F, H, J, L**; **Supplementary Figure S2B**). These results demonstrate that loss of *StPHR1* attenuates the global proteomic response to low-Pi stress, and that many Pi starvation-responsive proteins were not properly upregulated in its absence.

Under Pi-sufficient conditions, pairwise comparisons among the overexpression line, knockout line, and WT identified only small numbers of DEPs, with similar proportions of upregulated and downregulated proteins. No global proteomic perturbation was detected, indicating that altered *StPHR1* expression did not disrupt proteomic homeostasis under Pi-replete conditions. This result is consistent with the absence of significant growth differences among genotypes under normal phosphorus supply (**Figure 1**; **Supplementary Figure S2**). In the clustering analysis, low Pi-responsive proteins remained highly abundant in the *StPHR1* overexpression lines and were functionally associated with phosphate transport, acid phosphatase activity, secondary metabolism, and redox homeostasis. In contrast, their low Pi induction was largely abolished in the knockout lines, and their abundance did not differ significantly from that under Pi-sufficient conditions.

### 3.7 K-means clustering identifies *StPHR1*-dependent low Pi-responsive protein modules

K-means clustering of all quantified proteins was performed based on their abundance profiles across genotypes and Pi treatments. Nine clusters were identified, revealing the modular organization of the Pi-responsive proteome in potato (**Figure 9**). Proteins in Clusters 2, 3, and 5 showed stable abundance across samples, with minimal variation among genotypes and Pi treatments. These clusters were enriched in housekeeping proteins involved in basal metabolism and cellular structural maintenance. In contrast, proteins in Clusters 1, 7, and 9 displayed StPHR1-dependent Pi starvation-induced expression patterns. Their abundance was strongly increased by low-Pi stress in the *StPHR1* overexpression lines, moderately induced in WT, and nearly unchanged in the *StPHR1* knockout background. These proteins are therefore candidates for involvement in StPHR1-mediated low-Pi adaptation. Cluster 8 was the smallest cluster, comprising 140 proteins, but showed the highest overall abundance. Proteins in this cluster maintained high expression in the overexpression lines, suggesting that they may function as important nodes in Pi signaling.

**Figure 9:**
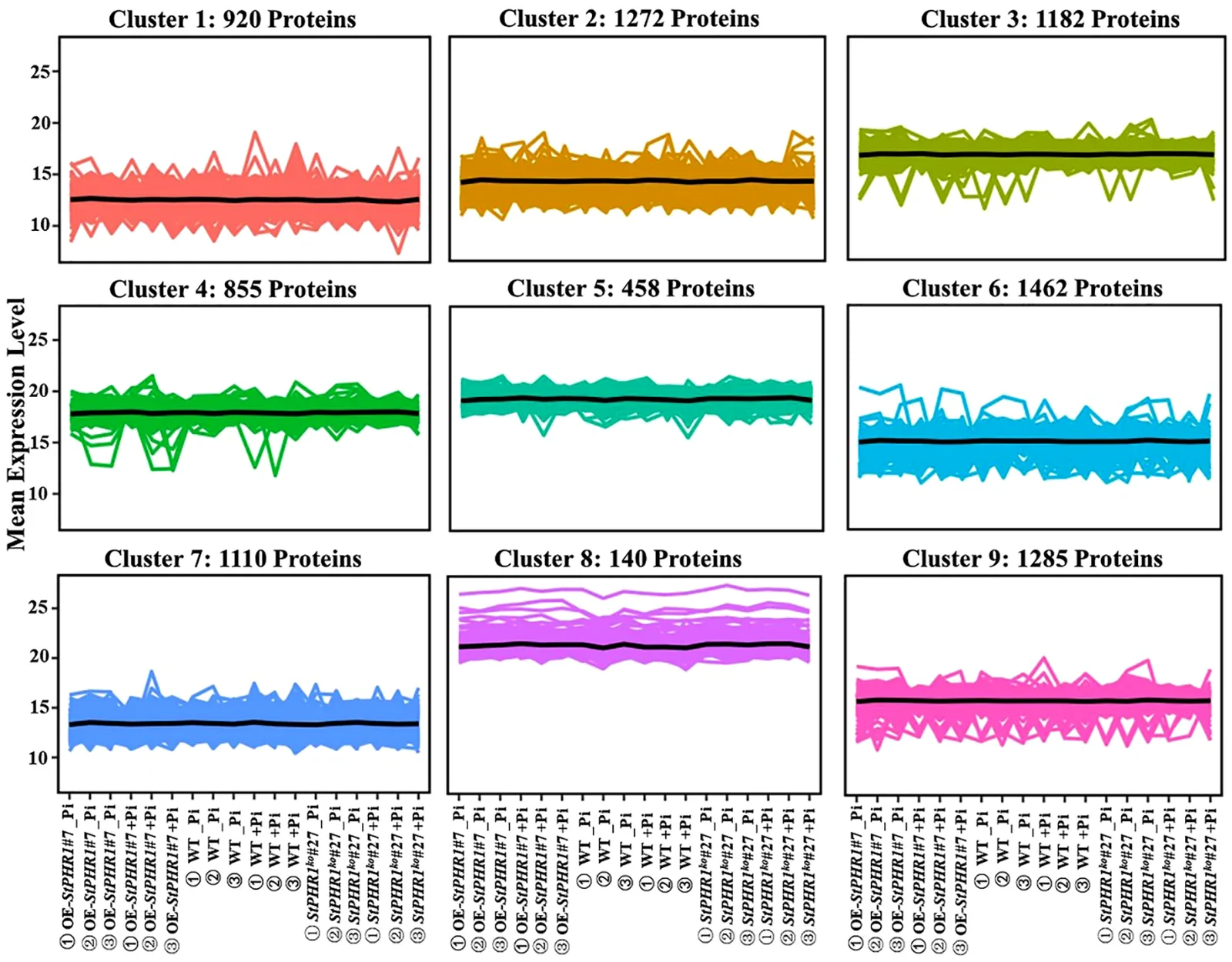
K-means clustering of abundance profiles for all quantified proteins. All quantified proteins were partitioned into nine clusters (Clusters 1–9) using the K-means algorithm based on their abundance patterns across samples. The number of proteins in each cluster is indicated in the corresponding subpanel title. Thin lines represent normalized abundance trajectories of individual proteins, and thick black lines indicate the mean abundance trend for each cluster. The x-axis corresponds to sample groups representing different genotype–Pi treatment combinations: the *StPHR1* overexpression line OE-*StPHR1*#7, WT, and the *StPHR1* knockout line *StPHR1*^ko^#27. Each genotype was analyzed under low Pi (−Pi) and Pi-sufficient (+Pi) conditions with three biological replicates per group. The y-axis represents normalized protein abundance.

### 3.8 GO enrichment analysis defines *StPHR1*-regulated low-Pi response pathways

GO annotation and enrichment analysis were performed for DEPs across the pairwise comparisons, and the results were visualized using bubble plots and WEGO classification charts. All enriched terms were assigned to biological process (BP), cellular component (CC), or molecular function (MF) categories (**Figure 2**; **Supplementary Figures S3**–**S5**). Under low-Pi conditions, DEPs between the *StPHR1* overexpression line and WT showed distinct enrichment across all three ontologies. Biological process terms included cellular catabolic processes, redox homeostasis, toxin detoxification, and secondary metabolite biosynthesis. Molecular function terms were dominated by oxidoreductase activity, alkyl/aryl transferase activity, and glutathione transferase activity. This profile indicates that *StPHR1* overexpression primarily affected redox, detoxification, and secondary-metabolic proteins under Pi limitation. Under Pi-sufficient conditions, DEPs between the overexpression line and WT were enriched at the cellular component level in plastids, chloroplasts, thylakoids, photosynthetic membranes, and photosystem-associated structures. Corresponding biological process terms were associated with photosynthesis and light harvesting. These results suggest an association between *StPHR1* overexpression and photosynthesis-related proteins under Pi-replete conditions.

Under low-Pi conditions, DEPs between the *StPHR1* knockout line and WT showed restricted functional enrichment. Biological process terms were concentrated in cell wall organization, phenylpropanoid metabolism, and cellular detoxification, whereas molecular function terms were dominated by peroxidase activity. The number of significantly enriched terms was much lower than in the overexpression comparison, indicating that loss of StPHR1 partially impairs activation of the Pi stress response (**Figure 2**). In the low-Pi versus Pi-sufficient comparison within the knockout line, DEPs were strongly enriched in lipid metabolism-related categories, including monoacylglycerol metabolism, diacylglycerol degradation, and phospholipase A1 activity, together with xenobiotic metabolic responses and negative regulation of seed germination. This pattern suggests that, without functional StPHR1, membrane-lipid remodeling was not fully engaged at the protein level and that lipid degradation and detoxification processes predominated instead. By contrast, the WT low-Pi versus Pi-sufficient comparison showed enrichment of classic phosphate starvation response terms, including ribosome biogenesis, RNA processing, and phosphoenolpyruvate carboxylase activity. This WT comparison serves as an internal validation of the Pi treatment regime.

### 3.9 WGCNA identifies *StPHR1*-dependent co-expression modules associated with low Pi phenotypes

Weighted gene co-expression network analysis (WGCNA) was used to partition all quantified proteins into co-expression modules, and hierarchical clustering dendrograms were constructed from the protein topological overlap matrix (TOM) (**Figure 10**; **Supplementary Figure S6**). Proteins were resolved into multiple discrete and highly interconnected modules. Proteins within the same module showed concordant abundance profiles, whereas connectivity between modules was lower than connectivity within modules, supporting the biological relevance of the module assignments (**Figure 10**). Colored bars along the dendrogram indicate module membership. Pearson correlation coefficients between module eigengenes and samples representing different genotype Pi treatment combinations were calculated, and correlation strengths and P values were visualized as a heatmap. Distinct genotype–treatment combinations showed specific module association patterns. Under low-Pi conditions, the *StPHR1* overexpression line was positively correlated with multiple modules enriched in phosphate starvation-responsive proteins, whereas the knockout line was negatively correlated with these modules. This pattern indicates that the abundance of proteins in these modules is associated with *StPHR1* activity; because WGCNA is a correlation-based approach, these associations do not by themselves demonstrate direct regulation (Langfelder and Horvath, 2008).

**Figure 10:**
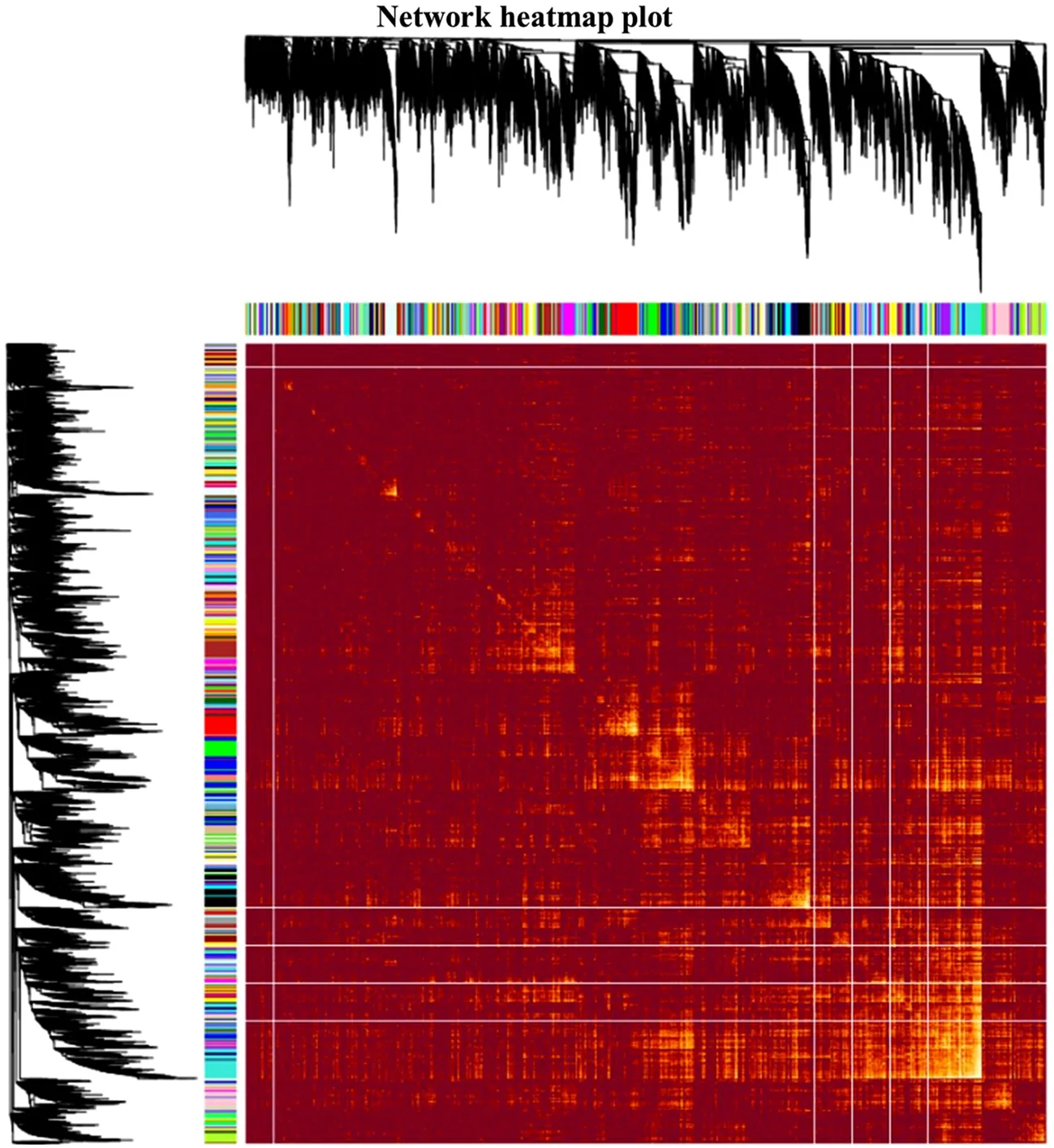
Topological overlap clustering heatmap of all quantified proteins. The heatmap was constructed from the abundance profiles of all quantified proteins to illustrate the modular architecture of the protein co-expression network. Each row and column corresponds to one protein. Color intensity reflects the topological overlap between paired proteins, representing similarity in abundance patterns and strength of co-expression connectivity. Proteins within the same co-expression module show highly concordant abundance patterns, whereas connectivity across modules is markedly lower. Dendrograms at the top and left show hierarchical clustering of all proteins, and the adjacent colored bars indicate module assignments. Data were derived from quantitative proteomic profiling of WT, OE-*StPHR1*#7, and *StPHR1*^ko^#27 under Pi-sufficient and low-Pi conditions.

Module–sample association patterns are consistent with a contribution of StPHR1 to Pi homeostasis (**Supplementary Figure S6**). Under low-Pi conditions, modules significantly positively correlated with WT were enriched in ribosome biogenesis, RNA processing, and phosphate metabolism, corresponding to the basal phosphate starvation response. This profile is consistent with global metabolic and transcriptional reprogramming under Pi stress in *Arabidopsis* (Plaxton and Tran, 2011). In addition to canonical Pi response pathways, modules specifically and positively correlated with the overexpression line were enriched in redox homeostasis, secondary metabolism, and detoxification proteins. This result suggests that *StPHR1* overexpression is further associated with increased abundance of stress-defense proteins under Pi deficiency. Under Pi-sufficient conditions, modules associated with photosynthetic machinery and plastid development were positively correlated with the overexpression line and negatively correlated with the knockout line, indicating that *StPHR1* also contributes to photosynthetic system maintenance when Pi is adequate. PHR proteins are known to bind P1BS *cis*-elements in target promoters in *Arabidopsis*, rice, and tomato (Rubio et al., 2001; Shi et al., 2021; Li et al., 2025); whether StPHR1 binds P1BS elements directly in potato remains to be tested. The core modules identified here provide a candidate set for identifying direct *StPHR1* target genes.

## 4 Discussion

This study characterizes the dual roles of StPHR1 in Pi homeostasis and tuber yield formation in potato by integrating reverse genetics with label-free quantitative proteomics. The results extend current understanding of PHR family functions beyond seedling-stage low-Pi responses and provide mechanistic insight into phosphate signaling in a tuber-bearing crop. Phylogenetic analysis of PHR homologs from 37 plant species positioned StPHR1 within a *Solanaceae*-specific clade together with SlPHR1 from tomato and CaPHR1 from pepper, distinct from other dicot and monocot lineages (**Figure 5A**). This topology is consistent with the evolutionary trajectory of MYB-CC transcription factors across plant taxa (Zhou et al., 2017). PHR proteins retain conserved MYB DNA-binding and coiled-coil domains from green algae to land plants, and their recognition of the P1BS cis-element underlies the conservation of the phosphate starvation response (Rubio et al., 2001). The *Solanaceae*-specific PHR1 clade suggests that PHR1 may have evolved regulatory features associated with the phosphate allocation demands of specialized storage organs, such as tubers and berries. This interpretation is consistent with the distinct phosphate utilization strategies reported in *Solanaceae* crops (Zhuomeng et al., 2024; Nasr Esfahani et al., 2025; Lin et al., 2025). Subcellular localization assays showed that StPHR1 is restricted to the nucleus (**Figure 5C**), as reported for AtPHR1 in *Arabidopsis* and OsPHR2 in rice. This localization is consistent with its function as a transcriptional regulator (Zhou et al., 2008; Puga et al., 2014). In *Arabidopsis*, SPX proteins bind PHR1 through Pi-dependent protein–protein interactions and repress the activation of downstream *PSR* genes (Puga et al., 2014; Yu et al., 2025). The nuclear localization of StPHR1 is compatible with a conserved SPX–PHR regulatory module in potato. It should be noted, however, that SPX-mediated regulation of StPHR1 in potato is inferred from model species and was not tested experimentally in this study. In addition, the root tip- and vascular-preferential promoter activity of *StPHR1* (**Figure 6**) parallels the root-biased expression of *OsPHR2* in rice (Zhou et al., 2008) and is consistent with a role for StPHR1 in root Pi accumulation and Pi homeostasis.

Genetic evidence from CRISPR/Cas9-mediated knockout and overexpression lines identifies *StPHR1* as a key contributor to low-Pi adaptation in potato (**Figure 3**). Under Pi-sufficient conditions, neither loss nor overexpression of *StPHR1* significantly affected seedling growth or internal Pi homeostasis, indicating that *StPHR1* activity is tightly controlled when phosphorus is abundant. This pattern is consistent with the SPX-mediated inhibition of PHR proteins under high-Pi conditions in *Arabidopsis* and rice (Bustos et al., 2010; Puga et al., 2014). Under low-Pi stress, *StPHR1* knockout lines exhibited impaired root elongation and severe shoot chlorosis, accompanied by significant reductions in whole-plant fresh weight and root and leaf Pi contents (**Figures 3B–E**). This low-Pi hypersensitive phenotype resembles those of the *Arabidopsis phr1* and rice *Osphr2* mutants, highlighting the functional conservation of *PHR1* across species (Rubio et al., 2001; Zhou et al., 2008). Conversely, *StPHR1* overexpression enhanced low-Pi tolerance: relative root length, biomass, and root and leaf Pi contents were all significantly higher than in WT (**Figure 4**). Similar positive effects of PHR overexpression on Pi accumulation and low-Pi tolerance have been reported in *Arabidopsis* and pepper (Nilsson et al., 2007; Cai et al., 2021). Genome-wide analyses in model plants show that PHR1 directly activates PHT1-family phosphate transporters, acid phosphatases, and numerous stress-responsive genes by binding P1BS elements in their promoters, thereby remodeling Pi acquisition and internal reutilization (Bustos et al., 2010; Zhang et al., 2014). In potato, loss of *StPHR1* appears to compromise the full deployment of PSR-associated proteomic responses under low-Pi conditions, reducing Pi accumulation in roots and limiting Pi supply to photosynthetic tissues; whether StPHR1 binds P1BS elements directly remains to be tested by electrophoretic mobility shift and chromatin immunoprecipitation assays. In addition, because whole-plant fresh weight was measured without separating root and shoot biomass, whether StPHR1 modulates the low-Pi-induced increase in the root-to-shoot ratio—a classic biomass-allocation response to Pi deficiency (Vance et al., 2003; Hermans et al., 2006)—remains to be determined.

Quantitative proteomic profiling indicates that StPHR1-dependent low-Pi responses are organized into distinct functional modules. K-means clustering showed that many proteins involved in basal metabolism and cellular architecture remained stable across genotypes and treatments, forming a basal module that sustains cellular homeostasis. In contrast, the induction of low Pi-responsive proteins depended strongly on StPHR1 activity: these proteins were highly upregulated in overexpression lines, whereas their Pi-dependent induction was largely abolished in knockout lines (**Figures 9**, **2**). This architecture, composed of basal homeostasis and stress-responsive modules, is consistent with the hierarchical model of the plant PSR. In this model, core transcription factors sense intracellular Pi status and regulate downstream genes, leading to changes in protein abundance, metabolic reprogramming, and stress adaptation (Zhang et al., 2014; Lin et al., 2025). Functional enrichment analysis showed that StPHR1-dependent DEPs were primarily associated with primary metabolic reprogramming, membrane-lipid remodeling, oxidative stress defense, and secondary metabolism. These profiles agree with phosphate stress proteome studies in *Arabidopsis* and rice (Lan et al., 2012; Pandey et al., 2025; Smith et al., 2026). In particular, the enrichment of proteins associated with phospholipid degradation and glycolipid biosynthesis supports the classic membrane-lipid remodeling strategy of Pi-starved plants, in which membrane phospholipids are replaced by non-phosphorus glycolipids to release Pi for essential processes (Nakamura, 2013). WGCNA further identified co-expression modules strongly correlated with genotype and Pi treatment (**Figure 10**; **Supplementary Figure S6**), providing a proteome-wide candidate pool for identifying key downstream nodes of StPHR1. It should be noted that DEP calling relied on nominal P values and fold-change thresholds without multiple-testing correction; the individual DEP results are therefore exploratory, and all proteomic conclusions were interpreted conservatively at the pathway level, where Benjamini–Hochberg-corrected enrichment was required; individual DEPs require independent experimental validation before functional roles are assigned to them. Similar network-based approaches have been validated in Pi stress studies of *Solanaceae* crops, including tomato (Langfelder and Horvath, 2008; Akash et al., 2021).

Agronomic trait analysis at maturity showed that *StPHR1* overexpression increased both tuber numbers per plant and average tuber fresh weight under standard cultivation conditions, producing a combined yield gain (**Figure 7**). This result extends the functional scope of PHR family proteins to tuber trait formation. Because the tuber trial was conducted under conventional, Pi-sufficient cultivation, this phenotype reflects an effect of enhanced Pi signaling under standard nutrient supply rather than improved low-Pi adaptation or phosphorus-use efficiency per se. Both direct and indirect mechanisms may contribute, and they are not mutually exclusive. A direct effect is plausible: StPHR1-mediated enhancement of Pi acquisition and translocation could increase internal Pi availability during stolon development and tuber bulking, processes with high Pi demand, given that Pi is required for energy metabolism, nucleic acid synthesis, starch modification, cell division in stolon tips, and tuber expansion (Fernie and Willmitzer, 2001; Nasr Esfahani et al., 2025). PHR transcription factors can also regulate phosphate transporter expression in storage tissues, facilitating directed Pi allocation to sink organs, and related mechanisms have been reported in developing rice grains and potato tubers (Rausch and Bucher, 2002; Wang et al., 2023). Indirect effects are equally plausible: improved phosphorus status can promote photosynthesis, carbon assimilation, and assimilate partitioning toward tubers, and tuber yield is additionally shaped by nitrogen metabolism and phytohormonal regulation (Hermans et al., 2006; Chea et al., 2024; Gong et al., 2025). Tuberization itself is governed by a multifactorial network that includes phytohormones, SHI/STY- and SRS-type transcription factors, ABA receptor components such as PYL proteins, and long non-coding RNAs (Gul et al., 2024; Yang et al., 2025; Ye et al., 2026); altered Pi status is therefore only one of several routes through which *StPHR1* overexpression could affect tuber traits. Two additional caveats apply. First, the proteomic data were obtained from roots of 14-day-old seedlings, whereas tuber traits were measured approximately 90 d after transplanting; molecular inferences linking StPHR1-dependent proteomic changes to tuber development therefore extrapolate across developmental stages and require validation at tuberization-relevant stages. Second, tuber quality traits—such as tuber phosphorus concentration, starch content, and soluble sugar content—were not measured in this study; given the documented influence of phosphorus supply on starch properties (Karim et al., 2007), such measurements represent an important next step in evaluating the agronomic value of *StPHR1* overexpression. These results provide an entry point for determining how phosphate signaling relates to tuber development and offer candidate targets for improving potato yield traits.

The potential costs of constitutive *StPHR1* overexpression also deserve consideration. In rice and *Brassica napus*, strong overexpression of PHR homologs caused excessive shoot Pi accumulation and Pi toxicity, resulting in growth retardation (Zhou et al., 2008; Ren et al., 2012). In *Arabidopsis*, PHR1 directly represses a subset of immune-related genes, and sustained activation of the phosphate starvation response can compromise defense against pathogens (Castrillo et al., 2017). Hyperaccumulation of Pi can also disturb the homeostasis of other nutrients, including iron and zinc (Khan et al., 2014). In the present study, no obvious growth penalty or nutrient imbalance was detected in the overexpression lines under Pi-sufficient conditions (**Figure 4**), which may reflect moderate overexpression levels; nevertheless, field evaluation of potential trade-offs—including pathogen susceptibility and micronutrient balance—will be essential before *StPHR1* overexpression can be considered for breeding. Fine-tuning *StPHR1* expression through promoter editing, rather than constitutive overexpression, may offer a route to capture the low-Pi tolerance benefit while avoiding these costs (Heuer et al., 2017).

Taken together, this study provides a comprehensive characterization of *StPHR1* function in potato through combined reverse genetics and quantitative proteomics. Relative to earlier work focused primarily on seedling-stage low-Pi responses, the present findings broaden the regulatory scope of PHR1 to include yield formation. As a tuber-bearing crop, potato differs from model plants such as Arabidopsis and rice in source–sink relationships governing Pi allocation and utilization. The StPHR1-dependent co-expression modules and DEPs identified here provide a data resource for dissecting lineage-specific mechanisms of efficient Pi regulation in tuber crops. The CRISPR/Cas9-derived homozygous knockout lines also provide useful genetic materials for future studies of potato phosphate signaling. Several limitations remain. First, direct binding of *StPHR1* to downstream target genes has not yet been verified by ChIP-seq or electrophoretic mobility shift assays, and its direct targets remain to be identified at the genome-wide level. Second, the regulation of StPHR1 by potato SPX family proteins is unresolved, and the conservation and lineage-specific features of the potato SPX–PHR module require further validation. Third, the tuber yield results are based on pot experiments; yield stability and phosphorus use efficiency under diverse field conditions remain to be tested. Fourth, although tissue Pi concentrations were quantified, total phosphorus uptake and phosphorus-use efficiency were not assessed. Accordingly, the specific contribution of StPHR1 to phosphorus-use efficiency remains to be fully elucidated. Fifth, genotype × Pi level interactions were not formally evaluated via factorial analysis. Phenotypic differences between genotypes are thus interpreted within each Pi condition, and formal validation of interaction effects will be performed in subsequent investigations. Multi-omics analyses combined with molecular validation are recommended for future work to map *StPHR1* target genes and characterize its interactions with SPX proteins. Field trials will be required to evaluate the potential of StPHR1 for Pi-efficient breeding (Heuer et al., 2017). Precise modulation of *StPHR1* expression through genome editing may also enable the development of elite alleles that improve low-Pi tolerance without causing Pi toxicity.

## 5 Conclusions

In summary, this study defines the evolutionary position, subcellular localization, promoter activity pattern, and biological functions of *StPHR1* in potato. *StPHR1* encodes a *Solanaceae*-associated MYB-CC transcription factor that localizes to the nucleus, and its promoter is preferentially active in root tips and vascular tissues. This spatial pattern is consistent with a role in root Pi accumulation and tissue-level Pi distribution. Genetic evidence from loss-of-function knockout and gain-of-function overexpression lines identifies StPHR1 as an important regulator of low-Pi adaptation in potato. *StPHR1* promotes root elongation, Pi accumulation, and biomass production under low-Pi conditions. It also increases tuber number and individual tuber weight, resulting in higher tuber yield under standard cultivation conditions. Quantitative proteomic analysis indicates that *StPHR1* coordinates the systemic low-Pi response by modulating proteins involved in metabolic reprogramming, membrane-lipid remodeling, and oxidative stress defense. Co-expression network analysis further identified modules associated with Pi starvation responses and photosynthetic development, providing candidate nodes for downstream functional studies. These findings refine the molecular framework of phosphate signaling in potato and provide genetic resources and candidate targets for improving low-Pi adaptation. Developing phosphorus-efficient cultivars is essential for reducing fertilizer input while maintaining crop productivity.

## Data Availability

The original contributions presented in the study are included in the article/**Supplementary Material**. Further inquiries can be directed to the corresponding authors.

## Supplementary material

Supplementary Figure 1 Pearson correlation heatmap of quantitative proteomic data across all samples.

Supplementary Figure 2 Abundance profiles of differentially expressed proteins in the *StPHR1* knockout line under low-Pi versus Pi-sufficient conditions.

Supplementary Figure 3 GO functional enrichment bubble plot of differentially expressed proteins in the *StPHR1* knockout line.

Supplementary Figure 4 GO functional classification of differentially expressed proteins (WEGO analysis).

Supplementary Figure 5 GO functional classification of differentially expressed proteins from three comparison groups (WEGO analysis).

Supplementary Figure 6 Heatmap of correlations between samples and co-expression modules.
