# Supplementary material for: The potato (Solanum tuberosum L.) transcription factor StPHR1 responds to low phosphate stress and regulates phosphorus accumulation
Source: Front Plant Sci. 2026 Oct 21;17:1975545. doi: 10.3389/fpls.2026.1975545 (PMC13730835; doi:10.3389/fpls.2026.1975545)
Supplement: Supplementary file 1 [file SupplementaryFile1.docx]

**The potato (*Solanum tuberosum* L.) transcription factor StPHR1** **responds to low phosphate stress and regulates phosphorus accumulation**

Feng Zhang^1, 2^, Huaijuan Gou ^2^, Yanlin An^2^, Wenlun Wang^2*^, Haiyan Zhang^3*^

**Figure S1** Pearson correlation heatmap of quantitative proteomic data across all samples.

**Figure S2** Abundance profiles of differentially expressed proteins in the *StPHR1* knockout line under low-Pi versus Pi-sufficient conditions.

**Figure S3** GO functional enrichment bubble plot of differentially expressed proteins in the *StPHR1* knockout line.

**Figure S4** GO functional classification of differentially expressed proteins (WEGO analysis).

**Figure S5** GO functional classification of differentially expressed proteins from three comparison groups (WEGO analysis).

**Figure S6** Heatmap of correlations between samples and co-expression modules.

**Figure S1** Pearson correlation heatmap of quantitative proteomic data across all samples. Rows and columns correspond to individual samples representing OE-*StPHR1*#7, WT, and *StPHR1*^ko^#27. Each genotype was analyzed under low Pi and Pi-sufficient conditions with three biological replicates per group. Values in each cell represent Pearson correlation coefficients between the global protein abundance profiles of paired samples. The color gradient from pale yellow to deep red corresponds to correlation coefficients from 0.90 to 1.00, with darker hues indicating greater similarity between samples.

**Figure S2** Abundance profiles of differentially expressed proteins in the *StPHR1* knockout line under low-Pi versus Pi-sufficient conditions. (A) Hierarchical clustering heatmap of differentially expressed proteins. Rows correspond to individual proteins, and columns correspond to samples from the *StPHR1*^ko^#27 line under low Pi and Pi-sufficient conditions, with three biological replicates per group. The color gradient from blue to red represents relative protein abundance from low to high. The dendrogram on the left shows protein clustering based on abundance patterns. (B) Volcano plot of differentially expressed proteins. The x-axis shows log_2_-transformed fold change in protein abundance, and the y-axis shows −log_10_-transformed P values. Red dots indicate significantly upregulated proteins, green dots indicate significantly downregulated proteins, and gray dots indicate proteins without significant abundance changes. The number of proteins in each category is annotated on the right side of the plot.

**Figure S3** GO functional enrichment bubble plot of differentially expressed proteins in the *StPHR1* knockout line. The plot shows enrichment results for the low-Pi versus Pi-sufficient comparison in the *StPHR1*^ko^#27 line. The y-axis lists enriched GO terms, and the x-axis indicates the number of differentially expressed proteins annotated to each term. Bubble size is positively associated with the number of enriched proteins per term. The color gradient from red to blue represents increasing P values (decreasing enrichment significance), with red indicating the highest significance. The legend on the right defines the protein-count scale for bubble size and the significance scale for the color gradient.

**Figure S4** GO functional classification of differentially expressed proteins (WEGO analysis). (A–D) Four pairwise comparisons: (A) low-Pi versus Pi-sufficient conditions in OE-*StPHR1*#7; (B) OE-*StPHR1*#7 versus WT under low-Pi conditions; (C) *StPHR1*^ko^#27 versus WT under low-Pi conditions; and (D) OE-*StPHR1*#7 versus WT under Pi-sufficient conditions. All terms were assigned to three Gene Ontology domains: biological process (BP, red bars), cellular component (CC, green bars), and molecular function (MF, blue bars). The y-axis shows log_2_-transformed counts of differentially expressed proteins annotated to each term, and the x-axis lists the GO terms.

**Figure S5** GO functional classification of differentially expressed proteins from three comparison groups (WEGO analysis). (A) Differentially expressed proteins in WT under low-Pi versus Pi-sufficient conditions. (B) Differentially expressed proteins in the *StPHR1*^ko^#27 line versus WT under Pi-sufficient conditions. (C) Differentially expressed proteins in the *StPHR1*^ko^#27 line under low-Pi versus Pi-sufficient conditions. All differentially expressed proteins were assigned to three Gene Ontology categories: biological process (BP, red bars), cellular component (CC, green bars), and molecular function (MF, blue bars). The y-axis represents log_2_-transformed counts of differentially expressed proteins annotated to each GO term, and the x-axis lists individual GO terms.

**Figure S6** Heatmap of correlations between samples and co-expression modules. Rows represent protein co-expression modules identified by weighted gene co-expression network analysis (WGCNA), each designated by a unique module color. Columns correspond to samples from different genotype–Pi treatment combinations, including OE-*StPHR1*#7, WT, and *StPHR1*^ko^#27. Each genotype was analyzed under low Pi and Pi-sufficient conditions with three biological replicates per group. Values in each cell denote Pearson correlation coefficients between the module eigengene and the corresponding sample and range from −1 to 1. The color gradient transitions from green (strong negative correlation) through white (no correlation) to red (strong positive correlation). Values in parentheses indicate the P value for each correlation.
